# Commentary: improving the health of neglected populations in Latin America

**DOI:** 10.1186/1471-2458-7-11

**Published:** 2007-01-23

**Authors:** Carlos Franco-Paredes, Danielle Jones, Alfonso J Rodríguez-Morales, José Ignacio Santos-Preciado

**Affiliations:** 1 Hospital Infantil de México, Federico Gómez, México D. F., México; 2 Department of Medicine, Emory University School of Medicine, Atlanta GA, USA; 3 Instituto Experimental José Witremundo Torrealba (Centro Trujillano de Investigaciones Parasitológicas José Witremundo Torrealba), Universidad de Los Andes, Trujillo, Venezuela

## Abstract

Neglected diseases encompass a group of pathologies that disproportionally affect resource-constrained areas of the world. In tropical and subtropical areas in Latin America, the vicious cycle of poverty, disease and underdevelopment is widespread. The burden of disease associated to neglected diseases in this region is mainly expressed through diseases such as malaria, dengue, intestinal parasitic infections, Chagas' disease, and many others. These maladies have burdened Latin America throughout centuries and have directly influenced their ability to develop and become competitive societies in the current climate of globalization. Therefore, the need for a new paradigm that integrates various public health policies, programs, and a strategy with the collaboration of all responsible sectors is long overdue. In this regard, innovative approaches are required to ensure the availability of low-cost, simple, sustainable, and locally acceptable strategies to improve the health of neglected populations to prevent, control, and potentially eliminate neglected diseases. Improving the health of these forgotten populations will place them in an environment more conducive to development and will likely contribute significantly to the achievement of the Millennium Development Goals in this area of the globe.

## Text

It is frequently implied in the global health literature that underdevelopment among human populations implies suffering. Indeed, living in the most resource-constrained settings with the worst possible health undeniably ensues human suffering. Thus, the life journey of the more than one billion people living in extreme poverty may occasionally provide them with some food, some maternal milk, dirty water; and often no opportunities for education and jobs; and consequently, a lack of health [1,2].

The current world order for people living in these conditions places them far away from the ethical and moral premise of the Universal Declaration of Human Rights and in a sharp evolutionary disadvantage [3,4]. In the case of Latin America, many millions of people die every year as a direct consequence of a vicious cycle of poverty, disease, and underdevelopment. In their struggle for survival, these individuals spend most of their existence gasping for breaths of humanity in the streets of major cities, rural areas, or shantytowns. The tragic reality is that the moral distance between the developed world and the forgotten populations in Latin America, may seem farther than the many million miles between the earth and the next planet. This distance can erode our moral identity enough to make the existence of the most vulnerable populations invisible: these people are "the nobodies".

**Figure 1:**
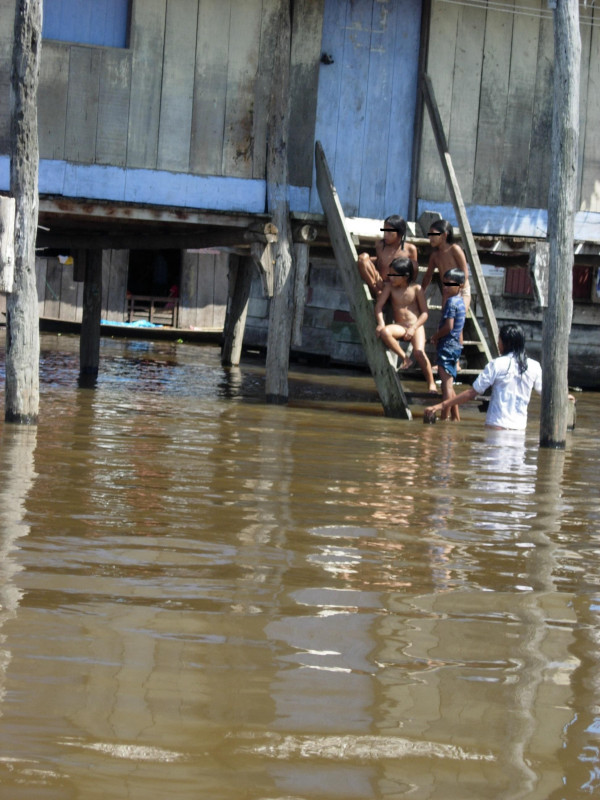
**Resource-constrained Belėn neighborhood in Iquitos, Peru**. People live in floating houses in flooded areas in a tributary river to the Amazon River. Stagnated Water is both their source of life (fish to eat, water to drink, cook, wash clothes and dishes, and to bathe with) as well as their source of demise by promoting diseases such as leptospirosis, dengue, malaria, intestinal parasitosis, bacterial gastroenteritis, and cholera.

Microbes have profoundly influenced the dynamics of human societies throughout the history of humankind by playing a key role as agents of natural selection [2,4]. Tragically, in this evolutionary struggle for survival, microbes have consistently abused the most impoverished human populations who dwell in unsanitary and crowded resource-constrained settings (Figure 1). This is why infectious diseases (most of which are preventable by simple cost-effective interventions) continue to impose an incalculable source of human misery and ever-rising death tolls. More than 95% of deaths due to infectious diseases occur in the developing world [2,4]. In addition, many infectious diseases lead to malnutrition, such that those who survive are left with a deficient nutritional status that entails an increased risk of infection, perpetuating an endless cycle of destitution.

Many diseases have historically been ignored or forgotten by leading public agencies, the pharmaceutical industry, and governments [3-6]. There has been a lack of investment for research and development for drugs to treat these diseases that affect mostly the poorest populations of the world since there is no revenue from these investments [6]. In addition, many governments have largely abandoned their own people suffering from various medical illnesses due to ignorance or due to a lack of resources to attack these maladies. Indeed, despite the grave impact of these diseases, the world's poorest patients are left completely marginalized.

This entrenched association between poverty and neglected infectious diseases is widespread in Latin America. More pervasive to this association is the fact that these neglected infectious diseases are often left out as public health priorities or their prevention and control programs are underfunded or are deemed nonsustainable. In tropical and subtropical areas of Latin America, the vicious cycle of poverty, disease and underdevelopment is imposed mainly by geographically restricted diseases such as malaria, dengue, and Chagas' disease. The pervasive health inequalities that prevail in Latin America manifest through these diseases, and provide crude illustrations of severe deprivation, misery, widespread pestilences. At times, this vicious cycle has even led to social disruption and civil strife (Figure 1) [4].

Thus, paving the way to improve the health status in this area of the world is a current priority. The challenge to achieve this public health goal is immense. Innovative approaches are required to ensure the availability of low-cost, simple, sustainable, and locally acceptable strategies to improve the health of neglected populations to prevent, control, and eliminate neglected diseases [3]. In this regard, integrative approaches using existing resources are promising in trying to alleviate some of the burden of disease in the most needed areas of the world. Holveck et al. [7] in this issue of the Journal should be commended for their efforts to streamline public health policies converting a totally inefficient old-fashioned vertical approach to public health to an inter-programmatic and inter-institutional horizontal comprehensive approach. This "holistic" approach seeks to attack the underlying social, economic, and environmental forces that are responsible for the expression of diseases of poverty.

This innovative perspective takes into account that poverty is the driving force of underdevelopment and bad health; and promotes community participation as a leading strategy to counteract the effects of disease determinants. The shifting paradigm proposed is moving from a disease-centered approach to a population health approach. These integrated approaches attempt to combine all programs (disease control programs), with the participation of various sectors (governments, businesses, civil society, etc.) to reach the overarching causes of poor health in these communities. As a hallmark of development, healthier and well-nourished children attend school, learn better and become productive adults able to contribute to economic growth and development in their communities. Basic integrated approaches that target children may include mass deworming programs, malaria control programs through distribution of mosquito nets, education about dengue personal protection measures, promoting attendance of children to primary school, clean water programs, and vaccination programs. Such activities represent a clear approach to promoting health and development of children as well as families.

There will be multiple barriers to the implementation of this approach. Substantial efforts will be required to perform operational research to evaluate the potential impact of this approach at a local, country, and regional level in Latin American countries; and to look at the social structures that contribute to illness. In order to produce long-term sustainable results, these programs should be carried out through the collaborative efforts of civil society, government, the Pan-American Health Organization (PAHO), and most importantly, the people of the affected communities.

The Millennium development goals provide an essential framework for the international community to approach the daunting task of eradicating extreme poverty and promote health and development [8]. These goals, particularly those that deal with the very basic human rights of clean water and the prevention of diseases by better nutrition, better sanitation, and vaccines, represent the highest health priority in this area of the globe. The efforts such as the one described in this paper by key members of PAHO offer a refreshing approach to identify opportunities to promote health and development in neglected areas of the Americas and perhaps elsewhere.

## Competing interests

The author(s) declare that they have no competing interests.

## Authors' contributions

CFP: conceived the idea of the editorial, and participated in its coordination and helped to draft the manuscript.

DJ: participated in drafting and editing the manuscript

ARM: participated in drafting and editing the manuscript

JIS: participated in drafting and editing the manuscript.

All authors read and approved the final manuscript

## Pre-publication history

The pre-publication history for this paper can be accessed here:


